# Athletic Identity and Performance Anxiety among University Athletes: Moderating Role of Perceived Coaching Styles

**DOI:** 10.11621/pir.2025.0105

**Published:** 2025-03-01

**Authors:** Fatima Mazhar, Muhammad Faran, Hamza Ameer, Sidra T.M Khan

**Affiliations:** a Bahria University, Islamabad, Pakistan

**Keywords:** athletic identity, perceived coaching styles, performance anxiety, university athletes, sports psychology

## Abstract

**Background:**

In the competitive world of emerging athletes, performance is crucial; in sports, it is essential. However, under the spotlight of competition, performance anxiety lurks as the unseen rival every athlete must conquer. Two important factors are fundamental to this dynamic—athletic identity and perceived coaching styles. Athletic identity becomes an athlete’s armor, while the coaching styles catalyze transformation. Their alliance can either elevate performance or diminish it.

**Objective:**

To investigate the relationship between athletic identity, perceived coaching styles, and performance anxiety among university athletes.

**Design:**

Using a correlational research design, 353 university athletes (191 men, 162 women, aged 18-25, M= 20.8, SD= 1.78) were recruited through non-probability purposive sampling from various universities. The athletic identity measurement scale (AIMS), leadership scale for sports (LSS), and sport anxiety scale-2 (SAS-2) were used to measure the constructs; the athletes also completed a demographic information sheet and provided informed consent.

**Results:**

The Pearson product moment correlation results indicated a signi" - cant negative correlation between athletic identity and performance anxiety, while perceived coaching styles were signi" cantly positively correlated with performance anxiety. The moderation analyses revealed that training and instruction, social support, democratic behavior, and positive feedback signi" cantly moderated the relationship between self-identity and performance anxiety. Additionally, training and instruction and autocratic behavior signi" cantly moderated the relationship between negative affectivity and performance anxiety.

**Conclusion:**

This study underscores the importance of understanding the dynamics between athletic identity, perceived coaching styles, and performance anxiety to optimize athletic performance and reduce performance anxiety among university athletes.

## Introduction

In the competitive world of emerging athletes, the significance of performance in sports cannot be understated; as Weinberg and Gould (2014) proposed, in the world of competitive sports, performance is everything. It is the driving force behind an athlete’s quest for greatness, and it is the ultimate measure of their abilities. Performance serves as the foundation upon which athletes build their achievements and accomplishments and is the result of their dedication, hard work, and persistent effort to achieve excellence (Chelladurai, 1980). The impact of anxiety in sports has long captivated the attention of coaches, athletes, and researchers. Performance anxiety can cast a shadow of doubt over even the most talented and dedicated athletes, creating a hurdle that can hinder their ability to showcase their true potential (Smith & Smoll, 1990). The research seeks to uncover the crucial role of perceived coaching styles in influencing performance anxiety among athletes and to change the nature of the relationship between athletic identity and performance anxiety.

The interaction between athletic identity, perceived coaching styles, and performance anxiety can be understood through the lens of self-determination theory. It can help to make sense of how athletes’ athletic identity and their perception of different coaching styles may impact levels of performance anxiety. The self-determination theory (Deci & Ryan, 2000) focuses on intrinsic motivation and three basic psychological needs — autonomy, competence and relatedness. In the context of sports, the fulfillment of these needs boosts athletes’ performance and wellbeing. When athletes feel autonomous, confident in their competence, and connected to others, they are intrinsically motivated, which increases engagement and decreases worry about performance (McDonough & Crocker, 2007; Vallerand & Losier, 1999). Athletic identity is the degree to which an athlete identifies with their sport, and it also plays a major role in motivation. Athletes who strongly identify with their athletic role are more likely to pursue sports for enjoyment and not as an obligation, enhancing their intrinsic motivation and performance. Coaching styles play a key role in the fulfilment of athletes’ psychological needs. A coaching style that supports autonomy leads to independence and positive reinforcement, which fulfills the needs for autonomy, competence, and relatedness. However, a style that controls autonomy pressurizes athletes, leading to burnout and performance anxiety by changing their motivation from intrinsic to controlled (Schutte & McNeil, 2015). This theory illustrates how athletic identity and coaching styles influence the performance and anxiety levels of athletes in sports. With the understanding of these dynamics, the athletes’ performance and overall wellbeing can be enhanced in the competitive sports environment.

The performance of athletes is influenced by both extrinsic and intrinsic factors (Almagro et al., 2020). Athletic identity, an intrinsic factor, plays a fundamental role in an athlete’s self-identity and connection to their sport. How they identify themselves as athletes, their sense of belonging, and their investment in their sport can impact their susceptibility to performance anxiety. On the other hand, coaching styles, as an extrinsic factor, can greatly influence an athlete’s performance and ultimately their level of performance anxiety. The way coaches interact with athletes, provide support, and create a positive and empowering environment can significantly alleviate or exacerbate performance anxiety. When coaches provide harsh feedback to the athletes who are afraid of failure and rejection, it can pose a threat to the athletic environment and provoke high levels of unpleasant emotions, which can increase the athletes’ level of performance anxiety (Passer, 1988; Roberts, 1986). On the other hand, athletes who perceive that their coaches support their efforts, enjoy sports more than those who have less favorable relationships with their coaches (Scanlan & Lewthwaite, 1986; Smoll et al., 1993).

Athletic identity is the degree of personal connection to sports (Edison et al., 2021). When a person truly embraces the athlete’s position and begins to identify with it, it starts to affect their participation in sport, their definition of themselves as athletes, and their pursuit of the sport; eventually, the meaning that that identification offers to their life is also affected (Haslam et.al, 2021). This was first conceptualized and rigorously examined by Brewer et al. (1993), who proposed that athletic identity can be defined as the extent to which an individual identifies with the athletic role. Ronkainen et al. (2016) suggested that athletic identity can be best understood as a multidimensional construct. Additionally, athletic identity can also function as a cognitive framework for coping mechanisms, information interpretation, and behavior that supports the athletic role (Heird & Steinfeldt, 2013). Brewer et al. (1993) explained athletic identity according to its social, cognitive, and behavioral components and in terms of self-identity, social identity, exclusivity, and negative affectivity. The self-identity of an athlete refers to the definition of who they are as a person; it builds their general self-concept (Chen et al., 2010). Social identity can be defined as the social role of an athlete, indicating that this identity is mostly generated through critiques by others, such as parents, coaches, teammates, and spectators and is affected by the perceptions of significant others (Brewer et al., 1993). Exclusivity refers to how heavily athletes rely on their athletic position relative to other roles, *e.g.*, as a friend, in their occupation, etc. (Brewer et al., 1993). Negative affectivity refers to the degree to which one feels negative emotions because of the undesired results in sports, *e.g.*, when an athlete is unable to train or compete (Brewer et al., 1993). According to Vallerand and Losier (1999), the motivational environment fostered by the coach also impacts the thoughts, feelings, and behaviors of athletes. An individual’s level of athletic identity becomes stronger with their level of motivation.

The role of the coach is critical for an athlete in terms of their performance, as the coach provides guidance, support, and structure for an athlete and helps the athlete to reach their maximum potential (Davis et al., 2018). Coaches use different styles when leading, motivating, and mentoring athletes, and these styles can vary according to the sport, the level of competition, and the athlete’s needs. Different coaching styles will have different effects on the athletes, and the coach must know which style to use in which situation and how these styles are being perceived by the athletes (O’Neil & Hodge, 2019). The concept of perceived coaching styles refers to the way in which coaches are perceived by the players in terms of how they coach and/or the environment in which they coach. Enhancing the athlete’s performance level is considered to be one of the most essential roles of a coach. Training and instruction can be viewed as a type of coaching behavior that focuses on assisting with intense and rigorous training; teaching athletes the skills and strategies of the sport; and explaining the dynamics among the teammates and organizing and arranging their activities to help them perform better. Democratic behavior can be viewed as a type of coaching behavior that is defined by the extent to which the coach permits athletes to be involved in decision making, practice methods, and game strategies and tactics. Autocratic behavior can be defined as a type of coaching behavior that is focused on independent decision making and emphasizes personal authority. Social support can be characterized as a type of coaching behavior in which the coach is concerned with the wellbeing of each and every athlete, a harmonious team environment, and friendly connections with the team members. Positive feedback can be defined as a type of coaching behavior which reinforces athletes, in which the coach recognizes and rewards their good performance (Chelladurai & Saleh, 1980).

The different coaching styles used by the coaches can greatly influence an athlete’s experience of anxiety. The way coaches interact with athletes and create a positive environment can significantly alleviate or exacerbate performance anxiety (Tsai & Chen, 2009). Performance anxiety is the mental state experienced when a person feels anxious and tense before, during, or after an event or performance. It is experienced by an individual who is apprehensive and afraid of failure and affects those who are typically susceptible to anxiety, particularly in scenarios involving public disclosure and competitive examination (Wilson & Roland, 2002). Sports performance anxiety can be typically defined as a negative emotional condition that arises in response to the stress of having to perform a task under pressure. It is experienced by athletes at any stage of performance and is frequently thought of as a normal reaction when an athlete’s performance and abilities are being evaluated (Smith & Smoll, 1990). According to Smith and Smoll (1990), sports-related anxiety is a distressing response that is linked with the stress of participating in sport and is made up of somatic components, such as the intensity of physical activity. It is a negative reaction that is commonly associated with the pressure of engaging in sports and involves cognitive factors, such as anxious thoughts and concerns. The difficulties faced by athletes in focusing on task-relevant cues, such as instructions by the coach, changing game situations, and cues related to performance, can also lead to performance anxiety (Smith et al., 2006).

## Literature Review

Numerous studies have been conducted in the field of sports psychology, including those on sports identity, perceived coaching styles, and performance anxiety in sports. Previous studies have explored athletic identity, perceived coaching styles, and performance anxiety, but have not examined the association between these three variables among Pakistani university athletes.

### Performance Anxiety

A research study conducted by Mercader-Rubio et al. (2023) investigated the relationship between levels of somatic anxiety, cognitive anxiety, and self-efficacy and the basic psychological needs of university athletes. The main findings revealed a consistent and significant relationship between self-efficacy and basic psychological needs. However, when considering cognitive anxiety, somatic anxiety, and autonomy, a similar relationship was not observed. Another study was conducted by Jooste et al. (2023) to explore the relationship between emotional intelligence and competitive anxiety in a sample of senior-level South African female hockey players. According to the findings, athletes who are more adept at controlling their own emotions as well as those of others are more likely to observe a decrease in cognitive anxiety and somatic anxiety. Moreover, research to explore sports-related anxiety in athletes was conducted by Ahmad and Safdar (2020), where the goal of the study was to examine the connections between competitive anxiety, goal orientation, and motivation in Pakistani domestic cricket players. According to the findings, ego orientation had a close connection with competitive anxiety when the athletes believed their abilities were sufficient to meet the demands of the circumstances.

### Athletic Identity

A study conducted by Hayes et al. (2023) examined the relationship between athletic identity and psychological distress and the moderating role of social support and self-compassion in this relationship among college athletes. The findings suggested that self-compassion and social support help to improve the negative effects on psychological wellbeing when athletic identity is disrupted. Ballesteros et al. (2022) conducted a study in which the goal was to examine the relationships between a student athlete’s academic and athletic identities and their overall wellbeing (*e.g.*, optimism and happiness levels) and athletic wellbeing (*e.g.*, satisfaction with one’s performance in a sport). Unexpectedly, athletic identity and sports wellbeing were found to be negatively correlated. Moreover, Haq and Kamran (2022) conducted research to investigate the relationship between athletic identity and life satisfaction in Pakistani athletes. The study’s goal was to find out whether athletic identity predicts life satisfaction among athletes or not. The results showed that there was no variation found in life satisfaction scores regardless of the type of sports or the level of competition.

### Perceived Coaching Styles

To understand how coaching styles are perceived by players and how achievement motivation is related to it among Pakistani basketball players, Atta et al. (2021) conducted extensive research on these athletes. The findings showed that a strong link was observed between the perceived coaching styles and achievement motivation. A study conducted by Keatlholetswe and Malete (2019) explored the relationship between coaching, perceived coaching styles, and team performance. This study looked at how players perceived coaches’ leadership styles, team environment, and the performance of their team throughout a soccer season. The results demonstrated that player opinions/perceptions of the coaches’ usage of all six leadership styles (democratic behaviors, positive feedback, training and instruction, situation consideration, social support, and autocratic behaviors) were predicted when the coaches rated themselves higher in technical efficacy. Another study was conducted by Kao and Tsai (2016) to investigate the relationship between transformational leadership and athletes’ satisfaction. The study also focused on investigating the mediating role of coaching competency between coaches’ transformational leadership and the athlete’s satisfaction (participation, performance, treatment, and training satisfaction). The results revealed that the coaches’ transformational leadership showed positive effects on coaching competency. Coaching competency was also proven to have a mediating effect between the positive effects of the coaches’ transformational leadership and the athlete’s satisfaction. The present research was conducted to address these gaps by investigating how athletic identity correlates with performance anxiety and how perceived coaching styles can play a role in changing the nature of this relationship in a sample of Pakistani university athletes.

## Hypotheses

1. There is a relationship between athletic identity (self-identity, social identity, exclusivity, and negative affectivity), perceived coaching styles (training instruction, democratic behavior, autocratic behavior, social support, and positive feedback behavior), and performance anxiety (somatic anxiety, worry, and concentration disruption) among university athletes.
2. Perceived coaching styles moderate the relationship between athletic identity and performance anxiety in university athletes.
3. Demographic variables (gender, experience, duration, type, and categories of played sport, etc.) are likely to have an impact on performance anxiety among university athletes.

## Methods

### Participants

The present study used a correlational (cross-sectional) research design. A sample comprising 353 athletes, calculated using G* power (Faul et al., 2009) and consisting of both men=191 and women=162 in the age range of 18-25 (*M*=20.8, *SD*=1.78) years, was recruited from different public and private institutes in Pakistan, using a nonprobability purposive sampling technique. The study included athletes who played their respective sports at the college level and were selected for the university’s sports team based on trials conducted by sports coaches. However, athletes who played at the international or national levels, alumni athletes, and physically handicapped athletes were excluded from the study.

### Procedure

The study plan was approved by the university’s research committee. Data collection involved visiting public and private universities, where the participants received informed consent forms and were briefed on the research. All 353 participants completed paper questionnaires with a 100 response rate. For the statistical analysis, the PROCESS macro was utilized for moderation analysis and interaction effects. The data were analyzed using IBM SPSS version 26 (Statistical Package for Social Sciences).

#### Questionnaires

*A thletic Identity Measurement Scale (AIMS)*. This scale was developed by Brewer et al. (1993) and is used to measure athletic identity. It comprises 10 items scored on a 7-point scale (1= strongly disagree, 7= strongly agree) and consists of four subscales (self-identity, social identity, exclusivity, and negative affectivity), which represent the social, cognitive, and affective aspects of athletic identity. According to the evidence obtained by Brewer et al. (1993), the Cronbach’s alpha calculation for the AIMS shows internal reliability, α= .93.

*Le adership Scale for Sports (LSS)*. This scale was developed by C helladurai and Saleh (1980) to measure the athletes’ preferences for particular leadership behaviors exhibited by their coaches and their perceptions of the actual coaching behaviors of their coaches. It can also be used to measure a coach’s perception of their own coaching behavior. This questionnaire comprises 40 items scored on a 5-point scale (1 = never, 5 = always) that consists of 5 subscales (training and instruction, autocratic behavior, democratic behavior, social support, and positive feedback behavior). The Cronbach’s alpha calculation shows internal consistency for training and instruction, α = .93; autocratic behavior, α = .79; democratic behavior, α = .87; social support, α = .86; and positive feedback behavior, α = .92.

*Sport Anxiety Scale 2 (SAS-2)*. This scale was developed by Smith et al. (2006) to measure cognitive and somatic trait anxiety in sport performance settings. It comprises 15 items scored on a 4-point scale (1 = not at all, 4 = very much) that consists of three subscales (somatic anxiety, worry, and concentration disruption). Each subscale consists of five items. The Cronbach’s alpha calculation for SAS-2 shows internal consistency, α = .91.

## Results

Initially, descriptive statistics and reliability analyses were conducted for demographic characteristics, athletic identity, perceived coaching styles, and performance anxiety. The Pearson product moment correlation coefficients were calculated to explore the relationships among these variables, including demographic factors. Finally, moderation analysis was performed to evaluate the moderating effect of perceived coaching styles on the relationship between athletic identity and performance anxiety.

The sample consisted of 353 university students with an average age of 20.8 years (SD = 1.78). Their mean CGPA was 3.2 (SD = .36). The athletes in the sample had an average playing duration of 15.99 months (SD = 9.84) at their current institute and a total professional playing experience of 51.78 months (SD = 16.62). They played an average of 2.63 hours per day (SD = 1.13). Among the participants, 191 (54.11%) were male, and 162 (45.89%) were female. A majority, 288 (81.59%), were from public universities, while 65 (18.41%) were from private institutions. Regarding living arrangements, 237 (67.14%) were day scholars, and 116 (32.86%) were hostel residents. Most (263, 74.5%) reported an urban background, while 90 (25.5%) came from rural areas. Sports participation varied: 142 (40.23%) played badminton, 70 (19.83%) cricket, 44 (12.46%) football, 34 (9.63%) basketball, 26 (7.37%) table tennis, 19 (5.38%) volleyball, 11 (3.12%) futsal, 4 (1.13%) chess, and 3 (0.85%) tennis. Most of the athletes (254, 71.95%) played team sports, 30 (8.5%) played individual sports, and 69 (19.55%) played both.

**Table 1:** Descriptive statistics of the demographic characteristics of the sample (N=353)

Variable	f	(%)	M	S D
Age (years)			20.8	1.78
CGPA			3.2	.36
Current Playing Duration (Months)			15.99	9.84
Professional Playing Experience (Months)			51.78	16.62
Daily Playing Hours			2.63	1.13
Gender				
Women	162	45.89		
Men	191	54.11		
Type of University				
Public	288	81.59		
Private	65	18.41		
Living Arrangement				
Hostel Resident	116	32.86		
Day Scholar	237	67.14		
Living Status				
Rural	90	25.5		
Urban	263	74.5		
Played Sports				
Basketball	34	9.63		
Badminton	142	40.23		
Football	44	12.46		
Volleyball	19	5.38		
Cricket	70	19.83		
Table tennis	26	7.37		
Tennis	3	0.85		
Chess	4	1.13		
Futsal	11	3.12		
Sports Type				
Individual sports	30	8.5		
Team sports	254	71.95		
Both	69	19.55		

*Notes: f=frequencies of demographic variables, = percentage, M= mean, and SD= standard deviations*

**Table 2:** Descriptive statistics and reliability analysis of athletic identity (social identity, self-identity, negative affectivity, exclusivity), perceived coaching styles (training and instruction, democratic behavior, autocratic behavior, social support, positive feedback), and performance anxiety (somatic anxiety, worry, concentration disruption) (N=353).

Variables	*k*	*M*	*SD*	Range		*α*
				Actual	Potential	
**Athletic Identity**	10	–	–	–	–	–
Social Identity	2	8.61	3.21	2–14	2–14	.70
Self-Identity	3	13.03	4.51	3–21	3–21	.75
Negative Affectivity	2	9.12	3.33	2–14	2–14	.76
Exclusivity	3	12.32	4.56	3–21	3–21	.79
**Perceived Coaching Style**	40	–	–	–	–	–
Training and Instruction	13	31.90	9.14	13–56	13–65	.82
Democratic Behavior	9	22.69	6.14	9–39	9–45	.71
Autocratic Behavior	5	14.49	4.27	5–25	5–25	.75
Social Support	8	22.16	5.50	9–40	8–40	.73
Positive Feedback	5	11.88	3.98	5–22	5–25	.72
**Performance Anxiety**	15	29.50	8.36	15–52	15–60	.84

*Notes: k= number of items, M = mean, SD = standard deviation, and α = Cronbach alpha reliability*

The Cronbach’s alpha reliabilities for the athletic identity subscales ranged from .70 to .79, and for the coaching style subscales, they ranged from .71 to .82; for performance anxiety, the reliability was .84.

The Pearson product moment correlation in *Table 3* shows that social identity was found to be negatively correlated with performance anxiety. Self-identity showed a negative association with training and instruction, positive feedback, and performance. Negative affectivity was found to be negatively correlated with training and instruction, democratic behavior, and positive feedback. Exclusivity was found to be negatively related to autocratic behavior, social support, somatic anxiety, and worry. Training and instruction, democratic behavior, autocratic behavior, social support, and positive feedback were found to be positively correlated with performance anxiety.

**Table 3:** Bivariate correlation between athletic identity (social identity, self-identity, negative affectivity, exclusivity), perceived coaching styles (training and instruction, democratic behavior, autocratic behavior, social support, positive feedback), and performance anxiety (somatic anxiety, worry, concentration disruption) (N=353)

Variables	*2*	*3*	*4*	*5*	*6*	*7*	*8*	*9*	*10*
**Athletic Identity**									
1. Social Identity	.67^***^	.31^***^	.35^***^	–.06	–.05	–.01	–.06	–.06	–.20^***^
2. Self-Identity	–	.37^***^	.43^***^	–.15^**^	–.095	–.014	–.07	–.15^**^	–.28^***^
3. Negative Affectivity		–	.32^***^	–.13^*^	–.17^**^	–.02	–.09	–.18^**^	–.06
4. Exclusivity			–	.00	–.06	–.10^*^	–.14^**^	.02	–.09
**Perceived Coaching Style**									
5. Training and Instruction				–	.72^***^	.27^***^	.54^***^	.72^***^	.36^***^
6. Democratic Behavior					–	.38^***^	.57^***^	.63^***^	.29^***^
7. Autocratic Behavior						–	.43^***^	.24^***^	.10
8. Social Support							–	.40^***^	.16^**^
9. Positive Feedback								–	.31^***^
**10. Performance Anxiety**									–

*p<.05, **p<.01, ***p<.001

**Table 4:** Regression analysis examining the interaction effect of athletic identity (social identity, self-identity, negative affectivity, exclusivity) and perceived coaching style (training and instruction, democratic behavior, autocratic behavior, social support, positive feedback) on performance anxiety (somatic anxiety, worry, concentration disruption) (N=353)

Variables	Performance Anxiety		
	*β*	*SE*	95 % CI
**Athletic Identity**	**–**	**–**	**–**
Social Identity	–.79	.60	[–.1.97, .39]
Self-Identity	–.34***	.09	[–.58, –.21]
Negative Affectivity	.93	2.43	[–5.71, .03]
Exclusivity	.41	2.56	[–4.63, 5.46]
**Perceived Coaching Style**	**–**	**–**	**–**
Training and Instruction	.25***	.04	[.01, .31]
Democratic Behavior	.32***	.06	[.19, .45]
Autocratic Behavior	.16*	.09	[.02,.35]
Social Support	.16*	.07	[.07, .31]
Positive Feedback	.46***	.10	[.25, .66]
**Interaction**			
Self-Identity x Training and Instruction	–.01*	.01	[–.03, –.001]
Self-Identity x Democratic Behavior	–.03*	.01	[–.05, –.001]
Self-Identity x Social Support	–.04**	.01	[–.07, –.01]
Self-Identity x Positive Feedback	–.04*	.02	[–.09, –.001]
Negative Affectivity x Training and Instruction	–.02*	.01	[–.05, –.001]
Negative Affectivity x Autocratic Behavior	.04*	.02	[–.01, .10]
*R^2^*	.259		
*F*	3.88***		

*p<.05,**p<.01, ***p<.001

The results of the moderation analysis showed that the main effect of athletic identity, including its subscale (self-identity), was found to be a positive predictor of performance anxiety. Furthermore, the main effects of perceived coaching styles, including its subscales (training and instruction, democratic behavior, autocratic behavior, social support, and positive feedback), were found to be positive predictors of performance anxiety.

**Figure 1:**
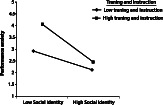
Interaction plot of self-identity and training and instruction on performance anxiety

**Figure 2:**
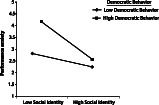
Interaction plot of self-identity and social support on performance anxiety

**Figure 3:**
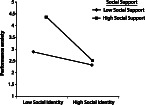
Interaction plot of self-identity and positive feedback on performance anxiety

**Figure 4:**
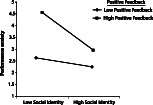
Interaction plot of self-identity and democratic behavior on performance anxiety

**Figure 5:**
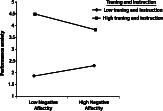
Interaction plot of negative affectivity and training and instruction on performance anxiety

**Figure 6:**
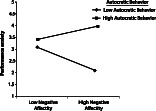
Interaction plot of negative affectivity and autocratic behavior on performance anxiety

For further analysis, the effects of the interactions between athletic identity (social identity, self-identity, negative affectivity, exclusivity) and perceived coaching styles (training and instruction, democratic behavior, autocratic behavior, social support, positive feedback) on performance anxiety (somatic anxiety, worry, concentration disruption) were examined; it was found that effect of the interaction between self-identity and training and instruction on performance anxiety was significant.

The interaction plots show that the relationship between self-identity and performance anxiety is increasingly negative at low, moderate, and high levels of training and instruction, social support, positive feedback, and democratic behavior, with a stronger negative relationship at high levels of these coaching styles. Additionally, the relationship between negative affectivity and performance anxiety is negative at high levels of training and instruction and positive at low levels. Conversely, this relationship is positive at high levels of autocratic behavior and negative at low levels.

**Table 5:** Independent sample t-test comparing performance anxiety (somatic anxiety, worry, concentration disruption) across genders (N=353)

	*Men*		Women				
Variables	(n=191)		(n=162)		t (351)	P	Cohen’s d
	M	SD	M	SD			
**Performance Anxiety**	26.81	7.82	32.68	7.87	7.00	.000	.74
Somatic Anxiety	8.57	2.97	10.69	3.14	6.53	.000	.69
Worry	9.44	3.43	11.26	3.22	5.11	.000	.54
Concentration Disruption	8.81	2.85	10.73	3.27	5.90	.000	.63
**Athletic Identity**							
Self–Identity	14.34	4.10	11.48	4.50	–6.22	.000	4.29
Social Identity	9.17	3.06	7.95	3.27	–3.62	.000	3.15
Exclusivity	13.12	4.53	11.37	4.42	–3.64	.000	4.48
Negative Affectivity	9.68	3.32	8.45	3.23	–3.49	.000	3.28
**Perceived Coaching Style**							
Training and Instruction	30.17	8.45	33.93	9.53	3.92	.000	8.96
Democratic Behavior	21.87	6.02	23.64	6.15	2.72	.007	6.08
Autocratic Behavior	14.40	4.33	14.59	4.21	.41	.679	4.27
Social Support	21.22	5.35	23.25	5.47	3.50	.000	5.41
Positive Feedback	11.15	3.72	12.74	4.10	3.81	.000	3.90

*Notes: M=mean, SD= standard deviation, p= significance level*

There were significant differences found in terms of performance anxiety and its subscales (somatic anxiety, worry, and concentration disruption) between men and women, as performance anxiety and its subscales were found to be higher in women compared to men, with a medium effect size. Furthermore, there were significant differences found in terms of athletic identity subscales (self-identity, social identity, exclusivity, and negative affectivity) between the men and women, as these variables were found to be higher in men compared to women, with a large effect size. The results showed that there were significant differences found in terms of the perceived coaching style subscales (training and instruction, democratic behavior, social support, and positive feedback); these variables were found to be higher in women compared to men, with a large effect size, whereas the results showed that there were non-significant differences in terms of autocratic behavior between the men and women.

## Discussion

The study aimed to investigate the relationship between athletic identity, perceived coaching styles, and performance anxiety among university athletes. The discussion included a presentation of the previous research that may have an impact on the findings.

Firstly, it was hypothesized that a relationship exists between athletic identity (social identity, self-identity, negative affectivity, exclusivity) and performance anxiety (somatic anxiety, worry, concentration disruption) among university athletes. The findings indicated that athletic identity was negatively correlated with performance anxiety. Specifically, social identity and self-identity were negatively correlated with somatic anxiety, worry, and concentration disruption, with self-identity being a negative predictor of performance anxiety. These results align with those of O’Connor et al. (2018), who found that a decline in athletic identity increased anxiety symptoms, and Hanton et al. (2003), who reported lower anxiety levels in athletes with a strong sense of self-identity and team identification. Exclusivity, defined as how strongly an individual relies on their athletic identity relative to other roles (*e.g.*, friend or occupation), was found to have a non-significant relationship with performance anxiety. This finding aligns with that of Masten et al. (2006), who stated that the weak definition of oneself in other roles does not significantly impact performance anxiety. Additionally, negative affectivity was negatively correlated with perceived coaching styles (training and instruction), supporting Chelladurai and Saleh (1980), who found that athletes with high negative affectivity perceived less benefit from coach instruction.

The study hypothesized that a relationship exists between perceived coaching styles and performance anxiety among university athletes. The findings revealed that positive feedback was positively correlated with worry and predicted higher performance anxiety; thus, they were consistent with the findings of John and Schweitzer (2021). Additionally, it was hypothesized that coaching styles would moderate the relationship between athletic identity and performance anxiety. The study found that high levels of training and instruction made the relationship between self-identity and performance anxiety negative; this supports the findings of Masten et al. (2006) and Price and Weiss (2000), who found that increased training and instruction enhances athletes’ skills and confidence by making them think they are capable enough and reduces their anxiety. High levels of social support also made the relationship between self-identity and performance anxiety negative, aligning with Bum and Shin (2015), who found that social support reinforces the athlete’s sense of belonging and validation and reduces cognitive anxiety before a game.

Furthermore, the study also found that high levels of positive feedback made the relationship between self-identity and performance anxiety negative, supporting Hong (2008), who found that positive feedback reduces cognitive anxiety (worry) because positive feedback allows athletes to embrace their identity with confidence and not fear, leading to a more positive self-concept. High levels of democratic behavior also made the relationship between self-identity and performance anxiety negative; this is consistent with Solstad (2018), who found that promoting athletes’ autonomy increases self-perceived ability and reduces fear of failure, thereby reducing performance anxiety. Lastly, it was hypothesized that demographic variables (gender, experience, duration, type, categories of played sport, etc.) are likely to have an impact on performance anxiety among university athletes. The findings of this study indicated that were differences found in performance anxiety between genders, where women had higher performance anxiety compared to men. This finding coincides with research conducted by Martinez-Gallego et al. (2022), which showed that female tennis players had higher levels of somatic anxiety than male tennis players. This could be due to the societal pressures women often face regarding performance, body image, etc., which can lead to heightened levels of performance anxiety. Another study also revealed that females reported higher levels of performance anxiety than males (Abrahamsen et al., 2008).

## Conclusion

There were significant relationships found between the subscales of athletic identity (social identity, self-identity, negative affectivity, exclusivity), perceived coaching styles (training and. instruction, democratic behavior, autocratic behavior, social support, positive feedback) and performance anxiety (somatic anxiety, worry, concentration disruption). The self-identity subscale emerged as a significant negative predictor of performance anxiety, while all the subscales of perceived coaching styles were found to be positive predictors of performance anxiety. Moreover, training and instruction, democratic behavior, social support, and positive feedback significantly moderated the relationship between athletic identity (self-identity) and performance anxiety. Additionally, training and instruction, as well as autocratic behavior, significantly moderated the relationship between athletic identity (negative affectivity) and performance anxiety. The findings underpin Deci and Ryan’s (2000) self-determination theory and can help to make sense of how athletes’ athletic identity and their perception of different coaching styles may impact levels of performance anxiety. A strong athletic identity increases intrinsic motivation by satisfying the psychological needs for autonomy, competence, and relatedness. The provision of autonomy support by coaches helps to foster a motivational climate that decreases performance anxiety and leads to optimal performance. On the other hand, the provision of controlling coaching styles undermines these needs and creates anxiety and diminished performance. With the understanding of these dynamics, coaches can not only promote confidence and strengthen athletes’ athletic identity but also adopt coaching styles that enhance their performance and overall wellbeing in the competitive sports environment. This study highlights the importance of coaches developing a coaching style that is democratic, supportive, and empowering rather than autocratic and controlling. By understanding the impact of coaching behavior on the performance of university athletes, coaches can adapt an approach to create a positive and motivating environment for university athletes and help them succeed in their athletic performance. The study can help raise public awareness about the significance of the presence of a sports psychologist among university athletes by shedding light on the prevalence of performance anxiety and its impact on university athletes. It emphasizes the significance of providing coach training programs that focus on effective coaching behaviors to enhance the ability to manage performance anxiety among university athletes.

The scope of future research could be broadened by including a wider variety of sports beyond the ones specified, such as cycling, marathon running, and weightlifting. Additionally, examining recreational sports alongside competitive ones could offer insights into how different contexts influence coaching dynamics and athletes’ anxiety levels. Future research could explore the impact of additional factors, such as motivation, academic stress, and nutrition, on university athletes’ performance and levels of performance anxiety.

## Limitations

The present study has several limitations that should be considered. The study included a specific set of sports. Therefore, the results may not be generalizable to university athletes playing other sports, such as recreational sports. The study aimed to understand university athletes’ performance in terms of their athletic identity and perceived coaching styles. There are various other factors that can affect the performance of university athletes that were not studied in the present research, such as motivation, academic stress, and nutrition.
